# Diagnostic potential of CDK1 and STAT1 in acute kidney injury associated with gastrointestinal cancers: a bioinformatics-based study

**DOI:** 10.3389/fmolb.2025.1522246

**Published:** 2025-01-22

**Authors:** Qiuwan Wei, Yiqing Shen, Yiren Tian, Yunzhi Ling

**Affiliations:** Department of Clinical Laboratory, Civil Aviation Shanghai Hospital, Shanghai, China

**Keywords:** CDK1, STAT1, bioinformatics, acute kidney injury, gastrointestinal cancers

## Abstract

**Introduction:**

Patients with gastrointestinal cancers are prone to acute kidney injury (AKI) due to treatment or disease progression, and current diagnostic methods exhibit insufficient sensitivity and specificity. This study aims to evaluate the potential value of CDK1 and STAT1 in the diagnosis of AKI in this patient population.

**Methods:**

A retrospective analysis was conducted on adjacent tissue, cancerous and the clinical data tissue from 150 gastrointestinal cancer patients treated at our hospital from May 2022 to May 2023. Differentially expressed genes (DEGs) associated with gastrointestinal cancer and kidney injury were identified through bioinformatics analysis. The expression of DEGs proteins in cancerous and adjacent tissues was assessed using immunohistochemical scoring (H scores). Patients were classified into AKI (n = 42) and non-AKI groups (n = 108) according to KDIGO AKI criteria. Univariate and multivariate logistic regression analyses were performed to investigate the influencing factors of AKI occurrence. Spearman correlation analysis was used to explore the relationship between DEGs and AKI biomarkers (Scr, BUN, MAU, and UA). The application value of DEGs in early diagnosis of AKI was evaluated using ROC curves.

**Results:**

Bioinformatics analysis identified CDK1, STAT1, COL1A2, and COL1A1 as DEGs related to AKI in gastrointestinal cancer. Immunohistochemical analysis revealed elevated H scores for CDK1, STAT1, COL1A2, and COL1A1 in tumor tissues. Univariate analysis showed no significant differences in age, sex, marital status, education level, monthly income, disease type, cancer stage, or tumor markers (CEA, CA242, CA50) between AKI and non-AKI groups (P > 0.05). However, the AKI group exhibited significantly higher levels of MAU, UA, and H scores for CDK1, STAT1, COL1A2, and COL1A1 compared to the non-AKI group (P < 0.05). Multivariate logistic regression confirmed that MAU, UA, CDK1, and STAT1 are independent risk factors for AKI in gastrointestinal cancer patients. Correlation analysis indicated a significant positive association between CDK1, STAT1, and AKI biomarker levels (P < 0.05). ROC curve analysis demonstrated that CDK1 and STAT1 possess high diagnostic value for early detection of AKI in patients with gastrointestinal cancer, with enhanced efficacy when used in combination.

**Conclusion:**

CDK1 and STAT1 serve as early diagnostic indicators for the occurrence of AKI in gastrointestinal cancer patients.

## 1 Introduction

Gastrointestinal cancers, including esophageal, gastric, and colorectal cancers, represent a significant proportion of malignant tumors globally, contributing notably to cancer-related mortality (Kanno et al., 2023; Ai et al., 2023). According to data from the Global Burden of Disease Study 2021, the incidence and mortality rates of gastrointestinal cancers in Asian countries increased significantly between 1990 and 2021. In 2021 alone, approximately 5 million new cases were reported, accounting for 27% of the global cancer cases, and contributing to approximately 22% of cancer-related deaths worldwide (Singh et al., 2023). Additionally, in low-resource regions, over 60% of cases are diagnosed at advanced stages. The scarcity of medical resources exacerbates the challenges of disease management, increasing the health burden on patients and the economic pressure on healthcare systems (Jiang et al., 2024). Advances in treatment technologies, such as surgery, radiotherapy, and chemotherapy, have substantially extended patient survival (Li et al., 2023; Yan et al., 2022). However, alongside improved survival rates, these treatments are also associated with notable side effects. Among these, acute kidney injury (AKI) is a common and severe complication. In low-resource areas, its incidence can reach as high as 30%, significantly impacting patient prognosis (Andresen et al., 2023; Ueno et al., 2023). AKI not only severely diminishes the quality of life but may also necessitate adjustments or interruptions in treatment plans, thereby affecting disease control and long-term survival. Chemotherapy agents, such as cisplatin and fluorouracil, are commonly used in the treatment of gastrointestinal cancers, but their nephrotoxic mechanisms significantly elevate the risk of AKI. Research has demonstrated that cisplatin induces acute renal dysfunction by triggering oxidative stress and directly damaging renal tubular cells, while fluorouracil may impair renal function by disrupting metabolic pathways and microcirculation (Ye et al., 2021; Perazella and Rosner, 2022; Kwiatkowska et al., 2021). Furthermore, abdominal or pelvic radiotherapy damages renal microvascular structures, further accelerating the deterioration of renal function (Ueno et al., 2023; Cytryn et al., 2023). Existing studies have also identified baseline health conditions such as dehydration, hypotension, and increased renal burden, as key risk factors for AKI (Leung and Rajkumar, 2023). Although traditional serum and urine biomarkers, such as serum creatinine and blood urea nitrogen, play a role in evaluating AKI, they typically detect abnormalities only at later stages of kidney injury and are limited in sensitivity and specificity. Consequently, they cannot reliably reflect the early onset of AKI. Identifying more specific and sensitive molecular biomarkers for the early diagnosis and monitoring of AKI is therefore of critical clinical importance for the treatment and management of gastrointestinal cancer patients.

Bioinformatics analysis, as an effective tool for exploring the molecular mechanisms of tumors and their related complications, has been widely applied (Chen et al., 2022; Huang et al., 2024). Through high-throughput data analysis, bioinformatics can identify key genes and signaling pathways closely associated with diseases, providing a crucial basis for discovering new diagnostic and therapeutic targets (Cui et al., 2023; Shi et al., 2022). Moreover, bioinformatics is not limited to the analysis of single-omics data but also integrates multi-omics data (e.g., genomics, transcriptomics, proteomics, and metabolomics) to construct more comprehensive molecular mechanism networks, offering integrative solutions for disease diagnosis and personalized treatment (Qiu et al., 2025). Currently, systematic studies on early diagnostic molecular biomarkers for AKI associated with gastrointestinal cancers remain lacking. To address this research gap, this study proposes the hypothesis that bioinformatics-identified molecular biomarkers, such as CDK1 and STAT1, may serve as highly sensitive and specific early diagnostic markers for AKI. This hypothesis aims to support improved early detection rates of AKI and enhance clinical treatment outcomes for gastrointestinal cancer patients. This study utilized bioinformatics approaches to systematically analyze AKI-related gene expression data in gastrointestinal cancer patients, identifying potential molecular biomarkers closely related with AKI. Data screening and analysis revealed that CDK1 (cyclin-dependent kinase 1), STAT1 (signal transducer and activator of transcription 1), COL1A2 (collagen type I alpha 2 chain), and COL1A1 (collagen type I alpha 1 chain) exhibited significantly differential expression in AKI-associated gene expression datasets. CDK1, as a key regulator of the cell cycle, not only plays a vital role in cancer cell proliferation but may also be critical in renal cell stress responses induced by chemotherapy and radiotherapy (Yin et al., 2024). STAT1, an essential regulator of inflammatory response and immune modulation, might play a key role in chemotherapy-induced renal immune reactions (Fu et al., 2023). Meanwhile, COL1A1 and COL1A2, as major components of type I collagen, may reflect the biological characteristics of fibrosis and repair during the AKI processes (Zhang et al., 2020).

In summary, CDK1, STAT1, COL1A2, and COL1A1 identified through bioinformatics methods show promise as early diagnostic molecular markers and potential therapeutic targets for AKI in gastrointestinal cancer patients, offering valuable references for clinical treatment and personalized management.

## 2 Materials and methods

### 2.1 Reagents and instruments

#### 2.1.1 Reagents

The list of reagents used in this study can be found in Table 1.

**TABLE 1:** Reagents.

Reagent	Batch number, manufacturer, city, country
Creatinine (Scr) Assay Kit	HBP31723R, Huabang Biological Technology Co., Ltd., Shanghai, China
Urea Nitrogen (BUN) Assay Kit	YS10086S, Yaji Biotechnology, Shanghai, China
Microalbuminuria (MAU) Assay Kit	E-EL-H6018, Elabscience Biotechnology Co., Ltd., Wuhan, China
Uric Acid (UA) Assay Kit	E-BC-K016-M, Elabscience Biotechnology Co., Ltd., Wuhan, China
CEA Assay Kit	E-EL-H6047, Elabscience Biotechnology Co., Ltd., Wuhan, China
CA242 Assay Kit	JLC6731, Jingkang Biological Engineering Co., Ltd., Shanghai, China
CA50 Assay Kit	ELK 1811, Kelubio Biotechnology Co., Ltd., Wuhan, China
Diaminobenzidine (DAB) Staining Kit	P0203, Beyotime Biotechnology, Shanghai, China
CDK1	ab201008, Abcam, Shanghai, China
STAT1	A0027, ABclonal, Wuhan, China
COL1A2	A5786, ABclonal, Wuhan, China
COL1A1	A22090, ABclonal, Wuhan, China
Goat Anti-Rabbit IgG H&L	ab6702, Abcam, Shanghai, China

#### 2.1.2 Software and instruments

The names of the software and instruments used in the study can be found in Table 2.

**TABLE 2:** Software and instruments.

Software/Instrument	Batch number, manufacturer, city, country
Automated Biochemical Analyzer	BS-1000M, Mindray Bio-Medical Electronics Co., Ltd., Guangdong, China
Jvenn v2.1	Université d’Orléans, Orléans, France
GEPIA v3.0	Peking University, Beijing, China
ImagePro Plus 6.0 Image Analysis System	Media Cybernetics, Inc.,Rockville, Maryland, United States
SPSS 26.0 Software	IBM Corporation, Armonk, New York, United States

### 2.2 Study subjects

A retrospective analysis was conducted on clinical data, adjacent normal tissues, and tumor tissues from 150 gastrointestinal cancer patients admitted to our hospital between May 2022 and May 2023. Based on the occurrence of AKI post-surgery, patients were categorized into AKI (n = 42) and non-AKI (n = 108) groups according to KDIGO AKI diagnostic criteria. The study was approved by the hospital’s ethics committee, and all participants signed informed consent forms. All research data were anonymized prior to analysis and strictly adhered to the Declaration of Helsinki and local ethical guidelines.

Inclusion Criteria: (1) All patients met the diagnostic criteria for gastrointestinal cancer (Hintzen et al., 2021); (2) Age ≥18 years (this age threshold was selected to align with adult AKI diagnostic standards, as renal function parameters differ significantly in pediatric populations); (3) Patients with complete clinical data, including critical renal function indicators (e.g., serum creatinine [SCr] and blood urea nitrogen [BUN]) both preoperatively and postoperatively; (4) Patients meeting the KDIGO AKI diagnostic criteria (Matuszkiewicz-Rowinska and Malyszko, 2020); (5) Patients with recordable renal function indicators, such as SCr and BUN, within 48 h after admission and treatment.

Exclusion Criteria: (1) Patients with a prior diagnosis of chronic kidney disease (to avoid confounding the diagnosis of acute kidney injury); (2) Patients with missing preoperative or postoperative creatinine measurements; (3) Patients with pimary malignant tumors invading or metastasizing to the kidneys, ureters, bladder, or other organs (to eliminate direct tumor effects on renal function); (4) Patients with mental disorders that affect participation in the study (5) Patients with significant comorbidities (e.g., diabetes, hypertension); to reduce the impact of potential confounding variables on AKI incidence.

### 2.3 Bioinformatics analysis

Gene expression data relevant to this research were sourced from the Gene Expression Omnibus (GEO) database, which includes datasets for various gastrointestinal cancers such as liver, colorectal, gastric, esophageal, and pancreatic cancers. ① Differentially Expressed Genes (DEGs) Screening: GEO datasets for these five types of cancers were analyzed, and intersection analysis was performed using Jvenn v2.1 to identify DEGs. The analysis parameters were set to p-value <0.05 and log2 fold change >1 or < −1. ② Functional Analysis: The identified DEGs were subsequently compared with kidney injury-related DEGs from the GeneCards database. The Jvenn tool was used for this analysis to identify DEGs associated with kidney injury. ③ Correlation Analysis: The GEPIA v3.0 tool was utilized to analyze the correlation between the identified DEGs and early kidney injury biomarkers, such as Cystatin C, NGAL, NAG, and KIM-1. This analysis aimed to assess the diagnostic potential of these genes.

### 2.4 Research methods

#### 2.4.1 Data collection

Data were collected through an electronic medical record system, including: ① General information [such as gender, age, marital status, education level, and monthly income]; ② Clinical data [including disease type, treatment methods, disease duration, and cancer staging]; ③ Laboratory examination data [including renal function indicators: creatinine (Scr), blood urea nitrogen (BUN), microalbuminuria (MAU), and uric acid (UA)], as well as hematological tumor markers [CEA, CA242, CA50].

#### 2.4.2 Immunohistochemistry (IHC)

The expression of CDK1, STAT1, COL1A2, and COL1A1 in tissue samples from gastrointestinal cancers patients was assessed. Tissue samples were cut into 4 μm-thick sections, deparaffinized with xylene, and blocked with 3% H₂O₂. The sections were incubated overnight at 4°C with primary antibodies for CDK1, STAT1, COL1A2, and COL1A1. After washing, the appropriate secondary antibodies were applied, and the sections were incubated at 37°C for 2 h. Staining was performed using a diaminobenzidine (DAB) staining kit, followed by a brief counterstaining with hematoxylin for 30 s. Finally, the stained sections were examined under a microscope, and ImagePro Plus 6.0 software was used for image analysis.

The staining results were independently evaluated by two pathologists from our institution, and the IHC results were scored using the H-score method, defined as staining intensity multiplied by the percentage of positive cells. Staining intensity was classified as 0 (no staining), 1, 2, or 3 (light yellow, brown, deep brown). The proportion of positive cells was scored as 0, 1, 2, 3, or 4 (representing 0%, 1%–25%, 26%–50%, 51%–75%, and 76%–100%, respectively). This study was approved by the ethics committee of Civil Aviation Shanghai Hospital (Ethics Approval Number: MHYY-CL-20230509).

### 2.5 Statistical analysis

A *post hoc* power analysis was performed using G*Power software to assess whether the sample size (n = 150) was sufficient to detect significant differences in the primary outcomes of the study. The analysis was based on the effect size derived from the observed differences in key variables (e.g., CDK1 and STAT1 expression levels) between AKI and non-AKI groups. A power threshold of 80% (β = 0.2) and a significance level of 0.05 (α = 0.05) were used as criteria. Data analysis was conducted using SPSS version 26.0. Quantitative data conforming to a normal distribution are expressed as means with standard deviations ( $\bar{x}\;\pm \;s$ ), with comparisons between two groups made using t-tests. For data not following a normal distribution, results are presented as medians with minimum and maximum values (M [Q_min_, Q_max_]), and group comparisons were performed using non-parametric tests. Spearman correlation analysis was utilized to assess the relationship between CDK1, STAT1, and renal injury. Receiver operating characteristic (ROC) curves were generated to evaluate the sensitivity and specificity of CDK1 and STAT1 for the early diagnosis of renal injury. The optimal cutoff value was determined based on the Youden Index, which maximizes both sensitivity and specificity to identify the best threshold. Multivariate logistic regression analysis was performed to assess the independent predictive value of CDK1 and STAT1 after adjusting for other confounding factors. The dependent variable was a binary variable (e.g., AKI/no AKI), and the relationships between the independent variables (MAU, UA, CDK1, STAT1, COL1A2, COL1A1) and the dependent variable were evaluated. The results are expressed as odds ratios (OR) with 95% confidence intervals (95% CI). A p-value of <0.05 was considered statistically significant.

## 3 Results

### 3.1 General characteristics of patients with gastrointestinal cancer

This study included a total of 150 patients, aged 18–47 years, with a mean age of 61.37 ± 11.26 years. Among the participants, 94 were male (62.67%) and 56 were female (37.33%). Details regarding marital status, educational level, personal monthly income, disease type, treatment methods, disease duration, cancer stage, tumor markers (CEA, CA242, CA50), MAU, and UA are presented in Table 3.

**TABLE 3:** General characteristics of patients with gastrointestinal Cancer [ $\bar{x}$ ±s, n (%)].

Factor	Group	Factor
Age	18–50 years	60.92 ± 10.71
Gender	Male	94 (62.67%)
	Female	56 (37.33%)
Marital Status	Single	14 (9.33%)
	Married	119 (79.33%)
	Divorced	11 (7.33%)
	Widowed	6 (4%)
Education Level	High school or below	104 (69.33%)
	University or above	46 (30.67%)
Monthly Income	RMB 5000 or below	111 (74%)
	Above RMB 5000	39 (26%)
Disease Type	Hepatobiliary cancer	32 (21.33%)
	Colorectal cancer	61 (40.67%)
	Gastric cancer	37 (24.67%)
	Esophageal cancer	11 (7.33%)
	Pancreatic cancer	9 (6%)
Treatment Type	Surgery	43 (28.67%)
	Chemotherapy	35 (23.33%)
	Surgery + Radiotherapy/Chemotherapy	55 (36.67%)
	Radiotherapy	17 (11.33%)
Disease Duration	<1 year	102 (68%)
	1–3 years	30 (20%)
	>3 years	18 (12%)
Cancer Stage	Stage Ⅰ-Ⅱ	54 (36%)
	Stage Ⅲ-Ⅳ	96 (64%)
CEA (ng/mL)		13.29 ± 3.10
CA242 (U/mL)		108.63 ± 18.61
CA50 (U/mL)		118.56 ± 18.92
MAU (mg/L)		27.27 ± 4.62
UA (μmol/L)		382.10 ± 86.10

### 3.2 Bioinformatics analysis reveals DEGs associated with gastrointestinal cancer and kidney injury

**FIGURE 1:**
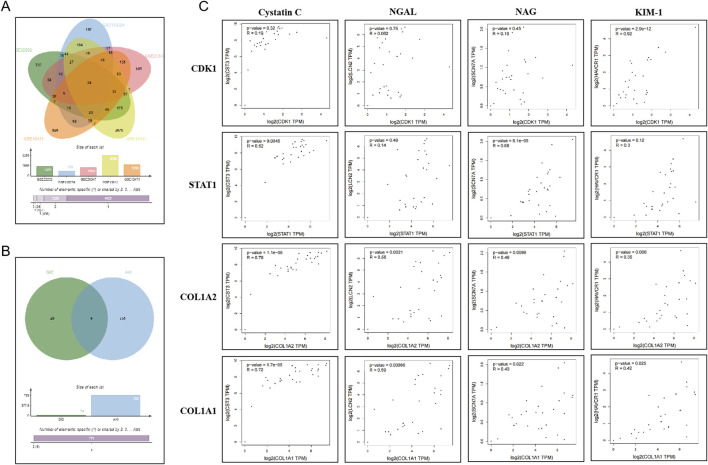
Bioinformatics Analysis of Genes Associated with Gastrointestinal Cancer and Kidney Injury. **(A)** Venn diagram illustrating the DEGs associated with gastrointestinal cancers. **(B)** Venn diagram showing the DEGs related to both gastrointestinal cancers and kidney injury. **(C)** GEPIA analysis demonstrating the correlation between overlapping genes and AKI biomarkers.

Using gene expression datasets related to gastrointestinal cancers from the GEO database—including liver cancer (GSE62232), colorectal cancer (GSE110224), gastric cancer (GSE20347), esophageal cancer (GSE13911), and pancreatic cancer (GSE15471)—intersection analysis was performed with selection criteria of P < 0.05, logFC > 1 or logFC < −1. A total of 24 DEGs were identified, including EPB41L4B, RRM2, ECT2, CCNB1, MAD2L1, PRC1, CDK1, CCNA2, DTL, NEK2, UBE2S, DLGAP5, KIF14, COL4A1, THY1, STAT1, LPCAT1, COL1A2, CEP55, CXCL10, LOC101928916///NNMT, BGN, SPP1, and COL1A1. Subsequently, these DEGs were compared with AKI-related genes from the GeneCards database, applying a filter for protein-coding genes. Further analysis identified CDK1, STAT1, COL1A2, and COL1A1 as DEGs associated with AKI. Correlation analysis between these DEGs and AKI biomarkers (Cystatin C, NGAL, NAG, and KIM-1) was conducted using the GEPIA tool, revealing significant correlations (p < 0.05) for CDK1, STAT1, COL1A2, and COL1A1, as illustrated in Figures 1A–C. These findings suggest that CDK1, STAT1, COL1A2, and COL1A1 may be involved in the AKI process associated with gastrointestinal cancers through mechanisms such as cell cycle regulation and inflammation, highlighting their potential as early predictive and diagnostic biomarkers for AKI.

### 3.3 Expression of CDK1, STAT1, COL1A2, and COL1A1 in gastrointestinal cancer patients

The expression levels of CDK1, STAT1, COL1A2, and COL1A1 in the tissues of gastrointestinal cancer patients were assessed using H-score analysis. The results indicated that, compared to adjacent non-cancerous tissues, the H-scores of CDK1, STAT1, COL1A2, and COL1A1 in cancer tissues were significantly elevated, with statistical significance (P < 0.05). See Table 4. These findings indicate that the expression levels of CDK1, STAT1, COL1A2, and COL1A1 are altered in gastrointestinal cancers, suggesting that they may be involved in the occurrence and progression of gastrointestinal cancers.

**TABLE 4:** Comparison of CDK1, STAT1, COL1A2, and COL1A1 expression in cancerous and adjacent non-cancerous tissues [M (Q_Min_, Q_Max_)].

Factor	Cancerous tissue	Para-cancerous tissue	z	P
	n = 150	n = 150		
CDK1	8 (1, 12)	3 (0, 6)	13.01	<0.0001
STAT1	6 (1, 12)	3 (0, 6)	12.23	<0.0001
COL1A2	8 (2, 12)	3 (0, 6)	13.21	<0.0001
COL1A1	8 (0, 12)	3 (0, 6)	12.89	<0.0001

### 3.4 Univariate analysis of general data between two patient groups

Based on the KDIGO AKI diagnostic criteria, all patients were categorized into AKI and non-AKI groups, followed by univariate analysis of their general data. The results revealed no significant differences between the two groups regarding age, gender, marital status, education level, personal monthly income, disease type, cancer stage, and tumor markers (CEA, CA242, CA50) (P > 0.05). Further analysis indicated that levels of urinary protein, blood uric acid, and key genes (CDK1, STAT1, COL1A2, and COL1A1) were significantly higher in the AKI group compared to the non-AKI group, with statistical significance (P < 0.05). See Table 5. The significant increase in CDK1, STAT1, COL1A2, and COL1A1 in the AKI group suggests that these genes may serve as early biomarkers for AKI and have clinical application potential in gastrointestinal cancer patients.

**TABLE 5:** Univariate analysis of general data between two patient groups [ $\bar{x}$ ±s, n (%), M(Q_Min_, Q_Max_)].

Factor		AKI group	Non-AKI group	t	P
		n = 42	n = 108		
Age		61.05 ± 8.49	60.87 ± 11.49	0.09	0.93
Gender	Male	29 (19.33%)	65 (43.33%)	1.02	0.31
	Female	13 (8.67%)	43 (28.67%)		
Marital Status	Single	5 (3.33%)	9 (6%)	1.10	0.78
	Married	31 (20.67%)	88 (58.67%)		
	Divorced	4 (2.67%)	7 (4.67%)		
	Widowed	2 (1.33%)	4 (2.67%)		
Education Level	High school or below	27 (18%)	77 (51.33%)	0.70	0.40
	University or above	15 (10%)	31 (25.83%)		
Monthly Income	RMB 5000 or below	33 (25%)	78 (52%)	0.63	0.43
	Above RMB 5000	9 (6%)	30 (20%)		
Disease Type	Hepatobiliary cancer	7 (6%)	25 (16.67%)	1.18	0.88
	Colorectal cancer	18 (12%)	43 (28.67%)		
	Gastric cancer	12 (8%)	25 (16.67%)		
	Esophageal cancer	3 (2%)	8 (5.33%)		
	Pancreatic cancer	2 (1.33%)	7 (6%)		
Treatment Type	Surgery	13 (8.67%)	30 (20%)	0.20	0.66
	Chemotherapy	9 (6%)	26 (17.33%)		
	Surgery + Radiotherapy/Chemotherapy	16 (10.67%)	39 (26%)		
	Radiotherapy	4 (2.67%)	13 (8.67%)		
Disease Duration	<1 year	29 (8.67%)	73 (48.67%)	0.04	0.98
	1–3 years	8 (5.33%)	22 (14.67%)		
	>3 years	5 (6%)	13 (8.67%)		
Cancer Stage	Stage Ⅰ-Ⅱ	13 (8.67%)	41 (27.33%)	2.04	0.15
	Stage Ⅲ-Ⅳ	29 (8.67%)	67 (44.67%)		
CEA (ng/mL)		13.35 ± 3.44	13.26 ± 2.98	0.16	0.87
CA242 (U/mL)		109.70 ± 19.90	108.21 ± 18.16	0.44	0.66
CA50 (U/mL)		119.25 ± 18.91	118.29 ± 19.00	0.28	0.78
MAU (mg/L)		29.36 ± 5.21	26.45 ± 4.11	3.6	<0.0001
UA (mmol/L)		409.78 ± 88.77	371.33 ± 82.97	2.50	0.01
CDK1		9 (3, 12)	7 (1, 12)	3.37	0.001
STAT1		8 (2, 12)	6 (1, 12)	3.16	0.002
COL1A2		9 (3, 12)	8 (2, 12)	2.31	0.02
COL1A1		9 (1, 12)	8 (0, 12)	3.35	0.001

### 3.5 Multivariate analysis of AKI in patients with gastrointestinal cancer

Based on the results of univariate analysis, significant differences between the AKI and non-AKI groups were observed in factors such as MAU, UA, CDK1, STAT1, COL1A2, and COL1A1. Therefore, these factors were included in the multivariate analysis. Using the occurrence of AKI in gastrointestinal cancer patients as the dependent variable (no = 0, yes = 1), multivariate logistic regression analysis was performed to explore the independent effects of these factors. The results indicated that elevated MAU, UA, CDK1, and STAT1 are independent risk factors for the development of AKI in gastrointestinal cancer patients (P < 0.05), as shown in Table 6. MAU and UA, as traditional biomarkers of kidney injury, are closely related to the risk of AKI development, consistent with their roles in kidney dysfunction. Furthermore, the elevation of CDK1 and STAT1 may suggest their potential involvement in the pathogenesis of gastrointestinal cancer-related AKI, although the specific mechanisms require further investigation. These results imply that the expression levels of CDK1 and STAT1 may provide valuable information for early clinical assessment of AKI risk, offering a reference for early diagnosis and intervention strategies.

**TABLE 6:** Independent risk factors for kidney injury in patients—multivariate logistic regression analysis.

Factor	β	Se	Wald χ^2^ value	p-value	Or	Or (95%CI)
Constant	12.093	2.277	28.197	0.000	178,637.576	
MAU	−0.134	0.053	6.291	0.012	0.875	0.989–1.000
UA	−0.006	0.003	4.608	0.032	0.994	0.788–0.971
CDK1	−0.233	0.089	6.804	0.009	0.792	0.665–0.944
STAT1	−0.238	0.090	6.964	0.008	0.789	0.661–0.941
COL1A2	−0.141	0.093	2.290	0.130	0.868	0.723–1.043
COL1A1	−0.088	0.095	0.844	0.358	0.916	0.760–1.104

### 3.6 Correlation analysis of CDK1 and STAT1 with AKI biomarkers

**FIGURE 2:**
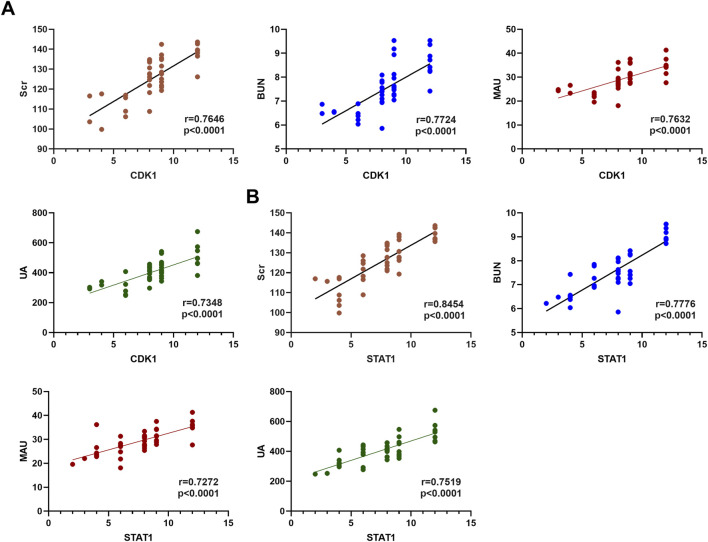
Correlation Analysis of CDK1 and STAT1 with Kidney Injury. **(A)** Spearman correlation analysis of CDK1 with AKI biomarkers. **(B)** Spearman correlation analysis of STAT1 with AKI biomarkers.

Based on the findings from the multivariate analysis, MAU, UA, CDK1, and STAT1 were identified as independent risk factors for AKI in gastrointestinal cancer patients. To further investigate the relationship between CDK1 and STAT1 with AKI biomarkers (Scr, BUN, MAU, and UA), correlation analyses were conducted. The results indicated that the correlation coefficients for CDK1 with Scr, BUN, MAU, and UA were 0.7797, 0.7114, 0.7632, and 0.7348, respectively, all demonstrating significant positive correlations. This suggests that CDK1 may play a critical role in the progression of AKI. Similarly, STAT1 showed correlation coefficients of 0.8454, 0.7776, 0.7272, and 0.7519 with Scr, BUN, MAU, and UA, respectively, further supporting its potential involvement in the pathological mechanisms of kidney injury. These findings imply that both CDK1 and STAT1 may have a significant pathological role in gastrointestinal cancer-associated kidney damage. See Table 7 and Figures 2A, B. These results suggest that the significant correlation between CDK1, STAT1, and AKI biomarkers may reflect their potential pathological roles in gastrointestinal cancer-related kidney injury.

**TABLE 7:** Correlation analysis of CDK1 and STAT1 with AKI biomarkers.

Factor	CDK1			STAT1		
	r	p	95% CI	r	p	95% CI
Scr	0.7646	<0.0001	2.649–4.478	0.8454	<0.0001	2.648–4.086
BUN	0.7724	<0.0001	0.1918–0.3687	0.7776	<0.0001	0.2313–0.3532
MAU	0.7632	<0.0001	0.9833–1.994	0.7272	<0.0001	0.9633–1.815
UA	0.7348	<0.0001	18.91–35.07	0.7519	<0.0001	19.54–32.34

### 3.7 Evaluation of CDK1 and STAT1 for early diagnosis of AKI

**FIGURE 3:**
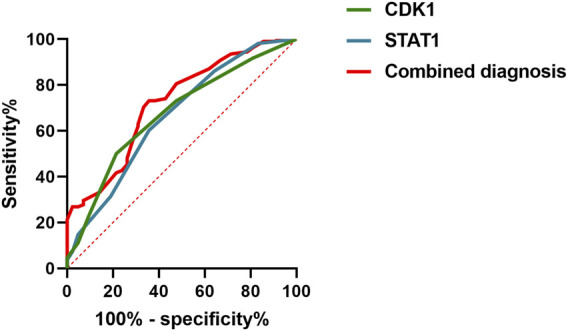
Roc curve evaluation of the diagnostic value of combined CDK1 and STAT1 for early detection of AKI.

ROC curve analysis demonstrated that the AUC for CDK1 was 0.6739, with a sensitivity of 50% and specificity of 78.57%. For STAT1, the AUC was 0.6630, with a sensitivity of 60.19% and specificity of 64.29%. When both markers were combined for diagnosis, the AUC increased to 0.7216, with a sensitivity of 73.15% and specificity of 64.29%, using a cutoff value of 0.3744. See Table 8 and Figure 3. These results suggest that the combined use of CDK1 and STAT1 has moderate diagnostic value and may have significant potential for the detection of early kidney injury. Particularly, the higher sensitivity (73.15%) could help identify kidney injury in its early stages, providing earlier opportunities for clinical intervention. Although the specificity is relatively low (64.29%), this combined method may still improve the accuracy of clinical diagnosis and reduce the likelihood of missed diagnoses. Therefore, the joint detection of CDK1 and STAT1 may provide valuable reference for early diagnosis.

**TABLE 8:** ROC curve analysis of CDK1 and STAT1 in the diagnosis of kidney injury.

Factor	AUC	Sensitivity%	Specificity%	Optimum cutoff value	95% CI
CDK1	0.6739	50%	78.57%	0.2857	0.5795–0.7684
STAT1	0.6630	60.19%	64.29%	0.2448	0.5633–0.7628
Combined diagnosis	0.7216	73.15%	64.29%	0.3744	0.6313–0.8119

Furthermore, this study is the first to identify CDK1 and STAT1 as independent risk factors for acute kidney injury in gastrointestinal cancer patients, positioning them as potential diagnostic biomarkers. This finding underscores their importance in early detection and offers new directions for clinical early intervention. By combining the diagnostic value of CDK1 and STAT1, this study provides new insights into the early identification and risk assessment of AKI in clinical practice, potentially having a positive impact on improving patient prognosis.

## 4 Discussion

Gastrointestinal cancers, including liver cancer, colorectal cancer, gastric cancer, esophageal cancer, and pancreatic cancers, are among the leading causes of cancer-related deaths globally. Despite significant advances in diagnostic and therapeutic methods, these cancers often involve various complications during treatment, with AKI being one of the most common (Jin et al., 2019). The occurrence of AKI can not only reduces the effectiveness of treatment but may also exacerbate the patient’s condition and even pose life-threatening risk (Ye et al., 2021). Traditional AKI biomarkers, such as Scr and BUN, typically show significant changes only in the later stages of kidney injury, and these indicators are also influenced by factors such as gender, age, and diet (Stevens et al., 2020). Therefore, identifying more sensitive and specific biomarkers for the early detection of AKI is of great clinical significance for improving the safety and quality of life of gastrointestinal cancer patients.

This study employed bioinformatics methods to analyze gene expression datasets related to digestive system cancers from the GEO database, identifying 24 common DEGs. An intersection analysis with known AKI-related genes from the GeneCards database revealed that CDK1, STAT1, COL1A2, and COL1A1 were significantly associated with AKI. These genes may play a key role in the opathological process of gastrointestinal cancers accompanied by AKI, providing new leads for further research. Previous studies have demonstrated that STAT1 plays an important role in the inflammatory signaling pathways of kidney injury (Fu et al., 2023), and this study further reveals its specific association with AKI in the context of gastrointestinal cancers, expanding its research scope in both nephrology and oncology (Yu et al., 2023). Bioinformatics analysis has become a powerful tool for exploring the molecular mechanisms of tumors and their related complications, widely applied in cancer research (Mustafa et al., 2024), offering new perspectives on the understanding of cancer and its complications. For example, Mustafa et al. (2024) explored the molecular mechanisms and emerging therapeutic strategies in pancreatic cancer, highlighting the critical role of bioinformatics in revealing cancer pathological processes and potential therapeutic targets. Additionally, Mir et al. (2016) research suggests that circulating autoantibodies in cancer patients have high specificity in cancer diagnosis, further emphasizing the importance of identifying molecular biomarkers for cancer diagnosis through bioinformatics methods.

Correlation analyses using the GEPIA tool indicated that CDK1, STAT1, COL1A2, and COL1A1 exhibit significant relationships with AKI biomarkers, such as Cystatin C, NGAL, NAG, and KIM-1 (p < 0.05). Notably, CDK1 showed a strong correlation with KIM-1 (r = 0.92), while STAT1 was correlated with Cystatin C and NAG (r = 0.52, r = 0.68), suggesting that these genes may play a central role in the biological functions associated with kidney injury. Furthermore, IHC results demonstrated that the H scores of CDK1, STAT1, COL1A2, and COL1A1 were significantly higher in cancerous tissues compared to adjacent normal tissues (p < 0.05). These findings suggest that these genes were also markedly elevated compared to non-AKI patients. This suggests that these genes may play an important role in the pathological process of gastrointestinal cancer-related kidney injury.

Univariate and multivariate analyses indicated further that CDK1 and STAT1 are independent risk factors for AKI in these patients, while COL1A2 and COL1A1, due to their primary role in maintaining extracellular matrix stability, did not show significant differences. The role of CDK1 in cell cycle regulation has been widely studied, and its high expression may be related to the activation of renal tubular cell proliferation following AKI to repair damaged areas (Cao et al., 2021). Additionally, STAT1 upregulation is closely associated with renal inflammation, and changes in its expression can reflect the severity of kidney injury (Xie et al., 2020). These results underscore the critical roles of CDK1 and STAT1 in the pathological process of kidney injury, highlighting their potential as biomarkers for early AKI detection and risk assessment. Moreover, Spearman correlation analyses revealed significant positive correlations between CDK1 and STAT1 with AKI biomarkers (Scr, BUN, MAU, and UA). This suggests that CDK1 and STAT1 may have a regulatory role in the pathological progression of kidney injury, further enhancing their potential as early diagnostic markers. Although this study accounted for key confounding factors such as age and gender through multivariate analysis, other potential confounders (such as underlying diseases and medication history) were not included in the analysis. Future studies could explore the impact of these additional factors on the occurrence of AKI, thus improving the clinical application value of these biomarkers.

In terms of diagnostic performance, ROC curve analysis showed that CDK1 had an AUC of 0.6739, with a sensitivity of 50% and specificity of 78.57%, while STAT1 had an AUC of 0.6630, with a sensitivity at 60.19% and specificity at 64.29%. When combined for diagnosis, the AUC increased to 0.7216, with a sensitivity of 73.15% and specificity of 64.29%. These results suggests that although CDK1 or STAT1 individually shows certain diagnostic efficacy for kidney injury, their sensitivity and specificity are not sufficient to fully meet clinical needs. However, their combined detection demonstrates higher diagnostic efficacy, indicating that the combined use of CDK1 and STAT1 could become an effective tool for predicting early kidney injury in gastrointestinal cancer patients.

In summary, this study identified CDK1 and STAT1 as significantly elevated in gastrointestinal cancer-related kidney injury through bioinformatics screening and experimental validation, suggesting their potential as diagnostic markers for early AKI. Their combined detection shows higher efficacy in early kidney injury diagnosis, indicating potential clinical value for assessing kidney injury risk. However, there are some limitations in this study. First, the findings rely primarily on gene expression data from public databases, which, while highly reliable, may not fully reflect genetic differences between populations. Second, the relatively small sample size (150 patients) may limit the generalizability of the results. Finally, the lack of longitudinal follow-up data prevents the assessment of long-term outcomes related to AKI. These limitations provide directions for future improvements, such as verifying these results through larger, multicenter clinical studies and exploring the prognostic role of CDK1 and STAT1 in AKI patients using long-term follow-up data. Despite these limitations, this study provides new molecular biomarkers for early AKI diagnosis in gastrointestinal cancer patients, particularly CDK1 and STAT1, whose combined detection shows potential for early kidney injury assessment and offers a solid foundation for clinical application. Especially in resource-limited regions, these biomarkers, through simple detection methods, may help improve early diagnosis rates, thus improving patient treatment and prognosis.

Future research could further validate the clinical reliability of CDK1 and STAT1 as biomarkers and explore their potential for targeted therapy. Evaluating the effects of CDK1 and STAT1 inhibition in animal models and assessing the impact of their combined application in clinical trials may provide new strategies for early diagnosis and treatment of AKI. Additionally, future studies should focus on the specific mechanistic roles of CDK1 and STAT1 in renal pathophysiology, particularly their involvement in the inflammatory response and repair processes in kidney injury. Combining CDK1 and STAT1 with other potential biomarkers to form a composite biomarker score could further enhance AKI diagnostic efficacy and early detection rates. These research findings could deepen our understanding of the molecular mechanisms underlying AKI and provide new directions for biomarker-based personalized treatment.

## 5 Conclusion

CDK1 and STAT1 may serve as early diagnostic indicators for kidney injury in patients with digestive system cancers. Their detection can reveal pathological changes in the initial stages of kidney injury, enabling early warning and timely intervention, ultimately contributing to improved patient outcomes.

## Data Availability

The datasets presented in this study can be found in online repositories. The names of the repository/repositories and accession number(s) can be found in the article/Supplementary Material.
